# Solvent-Free Synthesis of Highly Luminescent Solid-State Boric Acid-Assisted Carbon Dots for Stable and High-Contrast Latent Fingerprint Visualization

**DOI:** 10.1021/acsomega.6c08569

**Published:** 2026-10-20

**Authors:** Sema Karabel Ocal, Kevser Sahin Tiras, Evren Mutlugun

**Affiliations:** † Nanome Nanotechnology Co., Kayseri 38140, Türkiye; ‡ Department of Electrical-Electronics Engineering, Abdullah Gul University, Kayseri 38080, Türkiye; § Department of Physics, 52958 Erciyes University, Kayseri 38030, Türkiye; ∥ UNAM—Institute of Materials Science and Nanotechnology Bilkent University, Bilkent, Ankara 06800, Türkiye

## Abstract

The development of efficient, stable, and environmentally friendly fluorescent materials for latent fingerprint visualization remains a major challenge in forensic science. Carbon dots (CDs) have emerged as promising fluorescent nanomaterials owing to their low toxicity, tunable optical properties, and eco-friendly nature. However, their practical application in solid-state systems is often hindered by aggregation-induced quenching (AIQ), which can significantly suppress fluorescence emission. In this study, highly luminescent boric acid-assisted carbon dots (B-CDs) were synthesized through a simple, rapid, and solvent-free thermal process using citric acid, urea, and boric acid as precursors. Unlike conventional approaches, the developed method enables the direct formation of solid-state emissive carbon dot powders without requiring additional surface passivation, purification, or polymeric matrices. Structural and spectroscopic characterizations supported the successful formation of heteroatom-functionalized B-CDs that exhibit excitation-dependent photoluminescence and strong solid-state emission. The synthesized B-CDs exhibited a high solid-state photoluminescence quantum yield of 77.5%. The B-CDs showed strong short-term stability, retaining more than 95% of their initial PL intensity after 60 min exposure to 95% relative humidity and during short-term thermal treatment up to 90 °C. Long-term measurements showed approximately 57% PL retention after 5 days at 95% relative humidity and approximately 48% after 5 days of thermal aging at 50 °C, while approximately 92% of the initial PL intensity was retained after 5 days of continuous UV irradiation. The proposed solvent-free synthesis strategy offers a simple and potentially scalable route for producing strongly emissive solid-state carbon dots, highlighting their potential for latent fingerprint visualization.

## Graphical abstract

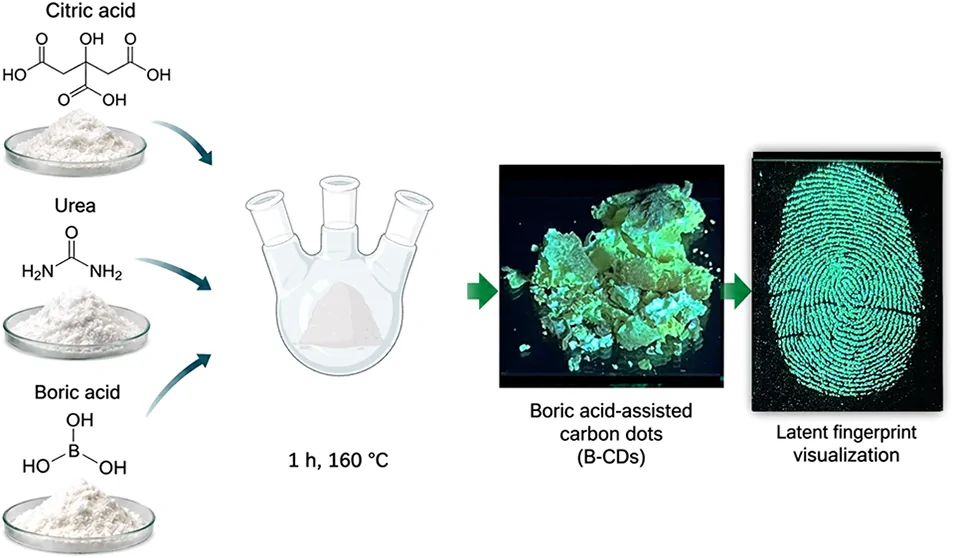

## 1 Introduction

Quantum dots (QDs) are nanoscale semiconductor materials, where the optical and electronic properties are strongly dominated by the quantum confinement effect.^1^ By controlling particle size, morphology, and composition, the absorption and emission properties of QDs can be finely tuned over a wide spectral range.^2^ Among the various types of quantum dots, carbon dots (CDs) have attracted considerable attention as promising substitutes for conventional heavy-metal-based nanocrystals due to their excellent biocompatibility, low toxicity, eco-friendliness, and facile preparation methods.^3^

In particular, their low-cost production from abundant carbon precursors makes them highly attractive for large-scale applications in optoelectronics,^4^ sensing,^5^ bioimaging,^6^ and anticounterfeiting.^7^ Nevertheless, despite their strong luminescence in dispersed systems, most CDs experience severe aggregation-induced quenching (AIQ) in the solid state.^8^ This limitation remains a major obstacle to the widespread use of CDs in solid-state luminescent applications.

Therefore, extensive efforts have been devoted to suppress AIQ through structural engineering and matrix-assisted confinement strategies. In previous studies, CDs have been incorporated into a variety of insulating and rigid host materials, including silica,^9,10^ starch,^11^ BaSO_4_,^12^ poly­(vinyl alcohol) (PVA),^13^ and polyhedral oligomeric silsesquioxane (POSS) frameworks,^14^ to spatially isolate the emissive centers and minimize interparticle interactions. In addition, inorganic salts and boron-containing compounds, such as CaCl_2_,^15^ NaCl,^16^ and boric acid^17–19^ have been employed as confinement matrices or crystallization media to reduce π–π stacking interactions and stabilize solid-state emission. These rigid matrices can effectively restrict particle aggregation and nonradiative energy transfer pathways, thereby improving the photoluminescence performance of CDs in the condensed state.

However, there is still a great need for simple, rapid, and scalable synthetic routes to solid-state emissive CDs, especially for forensic applications. In latent fingerprint visualization, fluorescent materials with strong solid-state emission and high surface affinity are essential for obtaining high-contrast ridge patterns and fine details. However, many previously reported CD systems rely on solution-based synthesis methods,^20^ polymer matrices,^21^ limiting their large-scale production and practical applicability.

In this regard, solvent-free synthesis strategies using boric acid as a rigid confinement matrix have attracted increasing attention for suppressing aggregation-induced quenching in solid-state CDs. During thermal synthesis, boric acid can spatially isolate CDs within the solid matrix, reducing π–π stacking interactions and preserving their luminescence properties without requiring additional surface passivation or polymer supports. Recent progress in CD-based latent fingerprint visualization has focused on improving solid-state fluorescence, image contrast, and the resolution of fine fingerprint features through heteroatom doping, surface modification, and incorporation of CDs into supporting matrices. Various CD-based fluorescent systems have recently been developed to improve fingerprint visualization on different substrates and enable the detection of detailed ridge characteristics. Despite these advances, many reported systems still involve solution-based synthesis, relatively long reaction times, additional processing steps, or supporting matrices, thereby motivating the development of simpler solid-state fluorescent materials that can be applied directly as fingerprint powders. Herein, B-CDs were synthesized via a simple, rapid, solvent-free thermal process and directly employed as highly luminescent powders for latent fingerprint visualization on various substrates. A key feature of this approach is the direct production of strongly emissive solid-state B-CD powders at a relatively low synthesis temperature (160 °C) within 1 h, without the need for solvents, additional purification, postsynthetic surface passivation, or polymeric matrices. The resulting B-CDs exhibit a high solid-state photoluminescence quantum yield (PLQY) of 77.5% and can be used directly as fluorescent fingerprint powders. This combination of a simple preparation route, strong solid-state emission, and direct powder applicability distinguishes the present system from many previously reported CD-based fingerprint materials.

## 2 Experimental Section

### 2.1 Materials

The citric acid (99.5–100.5%, Merck), urea (BioXtra, Sigma-Aldrich), boric acid (Emsure).

### 2.2 Synthesis of B-CDs

B-CDs were synthesized through a simple solvent-free thermal method. In a typical synthesis, citric acid (1.5 g), urea (0.33 g), and boric acid (1.3 g) were homogeneously mixed and transferred into a three-neck round-bottom flask. For comparison, pristine CDs were prepared under the same experimental conditions without adding boric acid. Before heating, the reaction system was evacuated using a mechanical vacuum pump, and the temperature was gradually increased to 50 °C to remove residual moisture and volatile species. The reaction temperature was then elevated to 160 °C under a nitrogen atmosphere and maintained for 1 h. During thermal treatment, the precursor mixture underwent simultaneous dehydration, polymerization, and carbonization, resulting in the formation of an expanded, foam-like porous structure. After naturally cooling to room temperature, the as-prepared solid product was gently ground into a fine powder using an agate mortar. A schematic illustration of the synthesis procedure is presented in Scheme 1.

**Scheme 1:**
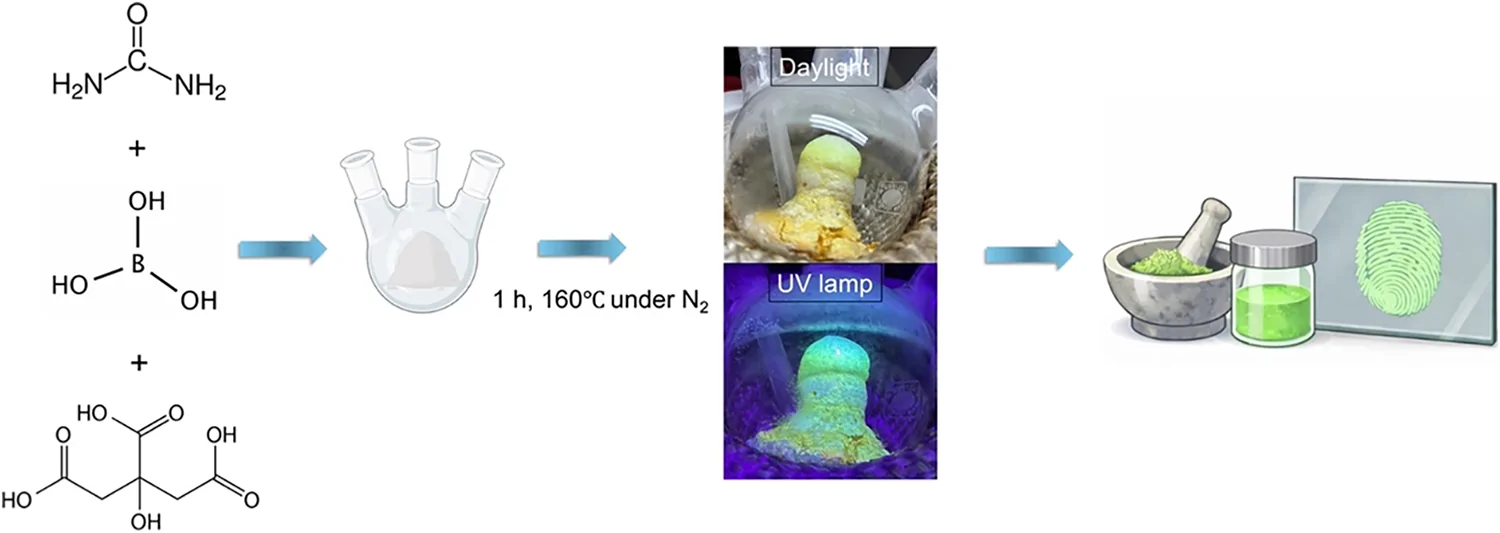
Schematic of the Synthesis Method and Formation Mechanism of CDs

### 2.3 Stability Tests

The stability of the synthesized B-CDs was systematically investigated under humidity, thermal, ambient air, and ultraviolet (UV) irradiation conditions. To evaluate short-term humidity stability, the samples were exposed to 95% relative humidity for 60 min, and the changes in photoluminescence (PL) intensity were monitored. Long-term humidity stability was additionally evaluated by maintaining the samples at 95% relative humidity for up to 5 days. Thermal stability was evaluated by depositing pelletized B-CD powders onto glass substrates and heating them on a hot plate at different temperatures (50, 70, 90, 120, and 150 °C) for 5 min. Long-term thermal stability was further assessed by maintaining the samples at 50 °C for 5 days. Ambient-air stability was evaluated by storing the B-CD powders at room temperature for 30 days. Photostability measurements were performed under continuous 365 nm UV irradiation (3 mW) at a sample-to-source distance of 20 cm. Short-term UV stability was evaluated over 60 min, while long-term photostability was monitored for up to 5 days.

### 2.4 Fingerprint Sample Collection

Latent fingerprints (LFPs) were collected from the right thumbs of volunteer donors following a standard deposition procedure. Prior to fingerprint deposition, the volunteers gently rubbed their fingertips across the forehead and nose regions to enhance the presence of natural sebaceous and sweat residues. The fingerprints were then transferred onto various substrates, including aluminum foil, ceramic surfaces, glass slides, metal, and plastic Petri dishes, using minimal pressure. For fingerprint visualization, the synthesized carbon dot powders were uniformly applied onto the fingerprint-containing surfaces. Excess powder was subsequently removed, and the developed LFPs were exposed to a gentle, low-pressure airflow for 30 s to eliminate weakly attached particles and reduce background interference. The fluorescence images of the developed fingerprints were then recorded using an optical microscope equipped with a fluorescence illumination source, enabling visualization of ridge details. For quantitative evaluation of fingerprint image contrast, five independent regions of interest (ROIs) were selected from representative fingerprint images obtained on glass, banknote, and aluminum foil surfaces. The fluorescence intensity of the ridge regions was compared with the local background intensity, and the ridge-to-background (R/B) signal ratio was calculated as R/B = *I*_ridge/*I*_background, where *I*_ridge and *I*_background represent the fluorescence intensities of the ridge and local background regions, respectively. The results are reported as mean ± standard deviation (*n* = 5).

### 2.5 Characterization

The X-ray diffraction (XRD) patterns were collected using a Bruker D8 Discover. Fourier transform infrared (FTIR) spectroscopy measurements were carried out with a Nicolet 6700 (Thermo Scientific). Transmission electron microscopy (TEM) images were acquired with a FEI Talos F200S operated at 200 kV. X-ray photoelectron spectroscopy (XPS) measurements were conducted using a PHI 5000 VersaProbe. Raman spectra were recorded using a WITec alpha300 M+ Raman spectrometer (Germany) equipped with a 532 nm excitation laser at room temperature. Optical characterization was performed using UV–visible (UV–vis), photoluminescence (PL), photoluminescence excitation (PLE), and time-resolved photoluminescence (TRPL) spectroscopies, along with PLQY measurements. The corresponding data were recorded using a Thermo Genesys 10S, a Cary Eclipse, a PicoQuant FluoTime 200, and a Quantaurus-QY (Hamamatsu), respectively. Bright-field optical images of the developed LFPs were captured using an Apple iPhone 13, utilizing its built-in color and contrast optimization and detail enhancement features. Fluorescence imaging was performed using a ZEISS Axio Observer.Z1/7 microscope equipped with an EC Plan-Neofluar 5×/0.16 M27 objective (NA = 0.16) under 488 nm excitation. Images were acquired at 2048 × 2048 pixels with a calibrated pixel size of 1.248 μm/pixel, corresponding to a field of view of approximately 2.56 mm × 2.56 mm.

## 3 Results and Discussion

In this study, B-CDs were synthesized via a solvent-free thermal process at 160 °C for 1 h under a nitrogen atmosphere, rather than by conventional solution-based synthesis. A simple, rapid, and environmentally friendly route was developed for the preparation of green-emissive B-CD powders exhibiting strong solid-state fluorescence and resistance to AIQ. The synthesis process is schematically illustrated in Scheme 1. Briefly, the precursor system consisted of citric acid as the carbon source, urea as the nitrogen source, and boric acid as the boron source and a key component influencing the solid-state environment of the CDs. During thermal treatment under a nitrogen atmosphere, the organic precursors underwent dehydration, polymerization, and carbonization, resulting in the formation of carbonaceous domains. Simultaneously, the boric-acid-derived environment may promote spatial separation of the newly formed CDs and reduce close interparticle interactions and π–π stacking. In addition, boric acid may modify the surface chemistry and emissive states of the CDs during carbonization. These combined effects are proposed to reduce aggregation-associated nonradiative relaxation and contribute to the enhanced solid-state photoluminescence of the B-CDs.

Figure 1 illustrates the effects of excitation wavelength and synthesis temperature on the PLQY of the CDs. As shown in Figure 1a, the CDs synthesized at 160 °C exhibited excitation-dependent PLQY, reaching a maximum value of 77.5% under 370 nm excitation. Figure 1b further shows that the PLQY increased with synthesis temperature and reached its maximum at 160 °C, indicating this as the optimum synthesis condition. The high solid-state PLQY of the B-CDs is attributed to the combined effects of structural confinement and surface-state modification induced by boric acid during thermal synthesis. The rigid boric-acid-derived environment may restrict close contact between neighboring carbon domains and reduce π–π stacking, thereby limiting aggregation-associated nonradiative relaxation. Meanwhile, the incorporation of B- and N-containing functionalities and the oxygen-rich surface chemistry, as supported by FTIR and XPS analyses, may modify and stabilize emissive surface states. The combined contribution of these effects is therefore considered responsible for the enhanced solid-state photoluminescence rather than physical confinement alone. Figure 2 further demonstrates the strong solid-state emission of the as-prepared B-CD powders, which appeared yellow under daylight and emitted intense green fluorescence under 365 nm UV irradiation. The achieved PLQY compares favorably with previously reported solid-state emissive CDs.

**Figure 1:**
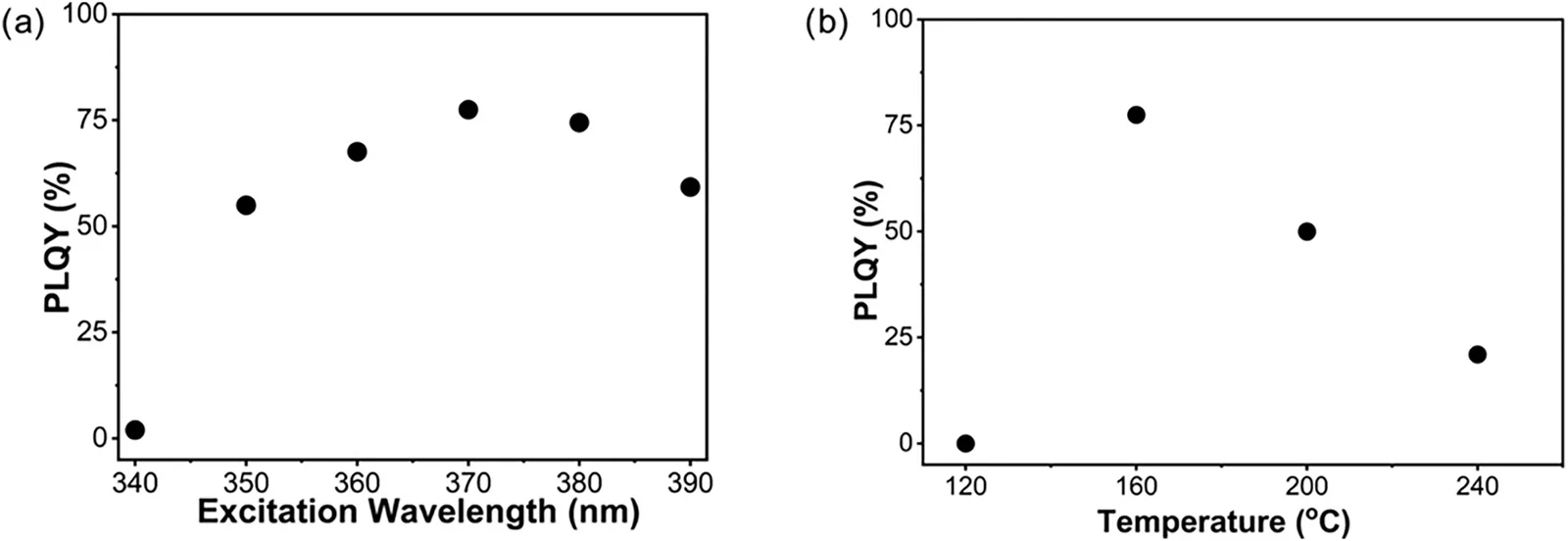
(a) Excitation wavelength-dependent PLQY of B-CDs synthesized at 160 °C. (b) Effect of synthesis temperature on the PLQY of B-CDs measured under 370 nm excitation.

**Figure 2:**
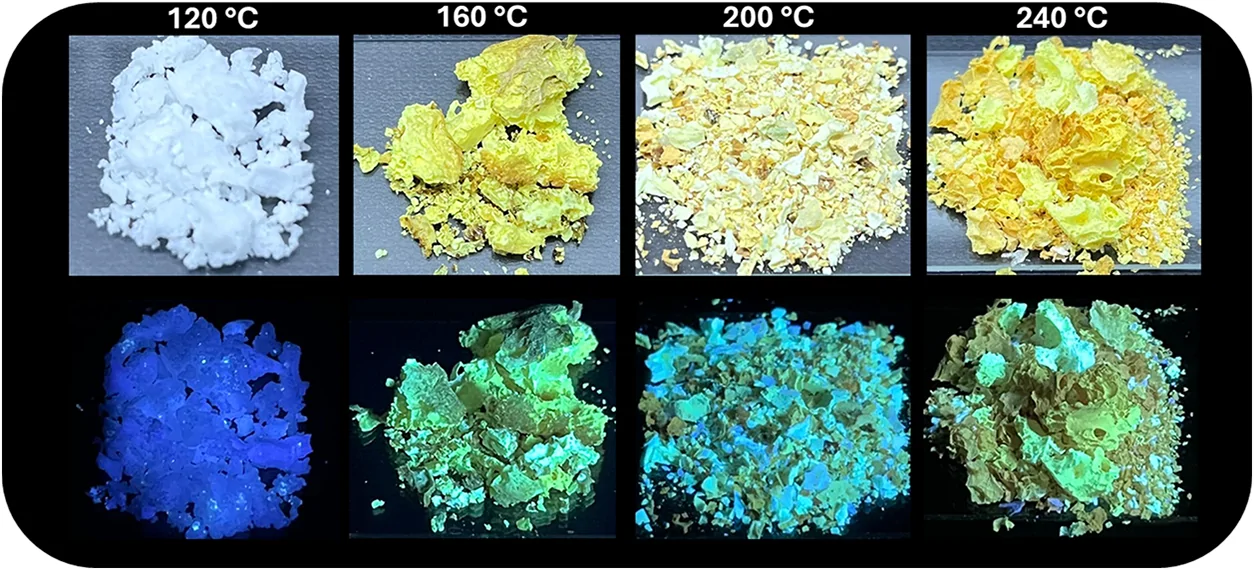
Photographs of B-CDs synthesized at different temperatures under daylight and UV light (365 nm).

For comparison, CDs synthesized in the absence of boric acid appeared black under both daylight and UV illumination and exhibited negligible solid-state luminescence (Figure S1). This observation supports the important role of boric acid in enhancing the solid-state photoluminescence of the B-CD system; however, the observed enhancement cannot be attributed exclusively to physical confinement, as boric acid may also influence the surface chemistry and emissive states of the CDs.^22^ To further investigate the excited-state dynamics and the influence of boric acid on the photophysical behavior of the CDs, TRPL measurements were performed for pristine CDs and B-CDs under 340 nm excitation. The decay curves were fitted using a three-exponential decay model, and the corresponding fitting parameters are summarized in Table S1. The amplitude-weighted average PL lifetime increased from 0.1749 ns for pristine CDs to 2.763 ns for B-CDs. This increase in the average PL lifetime is consistent with reduced nonradiative relaxation in the boric-acid-assisted system and provides additional support for the proposed contribution of the boric-acid-derived environment and surface-state modification to the enhanced solid-state photoluminescence. Nevertheless, the TRPL results are considered supporting evidence rather than definitive proof of an exclusively confinement-driven mechanism. The corresponding TRPL decay curves and comparative PL emission spectra of pristine CDs and B-CDs are presented in Figure S2a,b, respectively. The PL spectra show a pronounced enhancement in the emission intensity of B-CDs compared with pristine CDs, further supporting the important role of boric acid in enhancing the photoluminescence.

The as-synthesized CDs were obtained as a porous, foam-like structure composed of interconnected spherical domains. Following complete carbonization, highly luminescent CD powders exhibiting strong solid-state fluorescence were obtained directly without additional purification or postprocessing steps. To investigate the structural evolution and crystalline characteristics of the boric acid-assisted CDs, X-ray diffraction (XRD) analysis was performed (Figure 3a).

**Figure 3:**
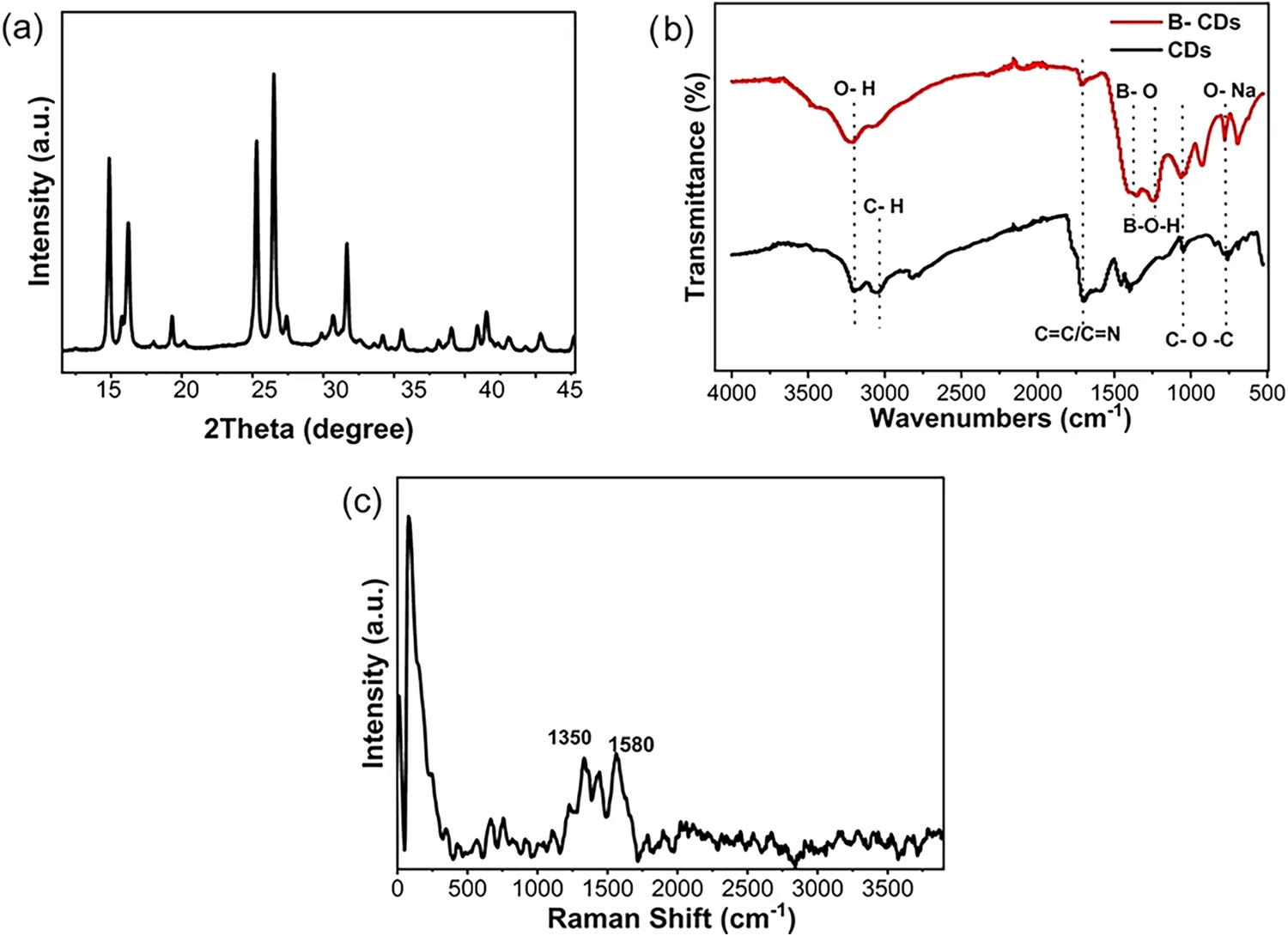
(a) XRD pattern, (b) FTIR spectrum, and (c) Raman spectrum of the synthesized B-CDs, revealing their structural characteristics, surface functional groups, and graphitic nature.

The XRD pattern of the synthesized CDs revealed diffraction peaks closely matching that characteristic of crystalline boric acid, confirming the presence of a boric acid matrix surrounding the CDs. The characteristic diffraction peaks located at 2θ ≈ 14.6°, 16.0°, and 31.6° were attributed to crystalline boric acid.^23–25^ Additional diffraction features likely originated from intermediate phases formed through interactions between boric acid and urea during thermal treatment.^25,26^ Furthermore, diffraction peaks observed at 2θ ≈ 19.6°, 23.0°, and 29.5° suggest the presence of carbonaceous domains and residual structural contributions associated with citric acid- and urea-derived species.^27^

The broad diffraction background alongside distinct crystalline peaks indicates that the obtained materials possess a mixed amorphous–crystalline nature, which is commonly observed in carbon dot systems synthesized from citric acid–based precursors.^28^ The amorphous component is primarily associated with disordered carbon cores, whereas the crystalline features are attributed to the surrounding boric acid matrix and precursor-derived phases. This hybrid structural nature is expected to play a crucial role in suppressing aggregation-induced quenching and preserving the solid-state luminescence of the synthesized CDs.

Functional groups originating from citric acid, urea, and boric acid were investigated by FTIR spectroscopy. The FTIR spectra of pristine CDs and B-CDs are presented in Figure 3b. In both samples, the broad absorption bands centered around 3250 cm^–1^ and 3050 cm^–1^ are attributed to O–H and C–H stretching vibrations, respectively, indicating the presence of hydroxyl and hydrocarbon surface functionalities. The absorption peak observed near 1750 cm^–1^ is assigned to carbonyl-related vibrations, mainly associated with C=O and/or C=N functional groups originating from surface oxidation and carbonization processes.^26,29,30^

Furthermore, the broad spectral region between 1900–1600 cm^–1^ observed in both samples can be attributed to vibrational modes associated with residual citric acid-derived structures and conjugated carbon frameworks formed during thermal treatment.^30,31^ The presence of boric acid in the B-CD structure is confirmed by characteristic B–O, B–O–H, and B–O–C vibrations located at approximately 1490 cm^–1^, 1250 cm^–1^, and 1196 cm^–1^, respectively, indicating successful interaction between boric acid and the carbon framework.^17,25^

In conventional carbon dots, strong π–π stacking interactions among sp^2^ carbon domains can promote aggregation-induced quenching in the solid state. In B-CDs, the boric-acid-derived environment may reduce close interparticle interactions and π–π stacking between neighboring carbon domains, thereby contributing to the preservation of solid-state emission. Moreover, variations in FTIR peak intensities indicate modifications in the surface functional groups during synthesis. In particular, the decreased intensity of oxygen-containing functional groups may be associated with dehydration and progressive carbonization of the precursor molecules, leading to changes in the surface chemistry and electronic structure of the B-CDs. Therefore, the enhanced solid-state photoluminescence is proposed to arise from the combined effects of reduced interparticle interactions and boric-acid-induced modifications of the carbon-dot surface and emissive states, rather than from physical confinement alone.^28^

The Raman spectrum of the synthesized B-CDs in Figure 3c exhibits two characteristic bands centered at approximately 1350 cm^–1^ and 1580 cm^–1^, corresponding to the D and G bands of carbon materials, respectively. The D band is associated with structural defects, disorder, and the breathing mode of sp^2^ carbon rings, while the G band originates from the in-plane stretching vibration of sp^2^-hybridized graphitic carbon atoms.^1^ The presence of both bands confirms the formation of a partially graphitized carbon framework containing abundant defect sites. The relatively pronounced D band suggests a high density of structural defects, likely due to the incorporation of boron species and the presence of oxygen-containing surface functional groups. These defect sites enhance photoluminescence by introducing emissive surface states while maintaining sufficient graphitic domains for efficient electronic delocalization.

The FTIR findings were further validated by X-ray photoelectron spectroscopy (XPS) analysis, which provided detailed insight into the elemental composition and surface chemical states of the B-CDs and pristine CDs. As shown in Figure 4a, the survey XPS spectrum revealed the presence of characteristic B 1s, C 1s, N 1s, and O 1s signals, confirming the successful incorporation of boron alongside carbon, nitrogen, and oxygen into the synthesized structure.

**Figure 4:**
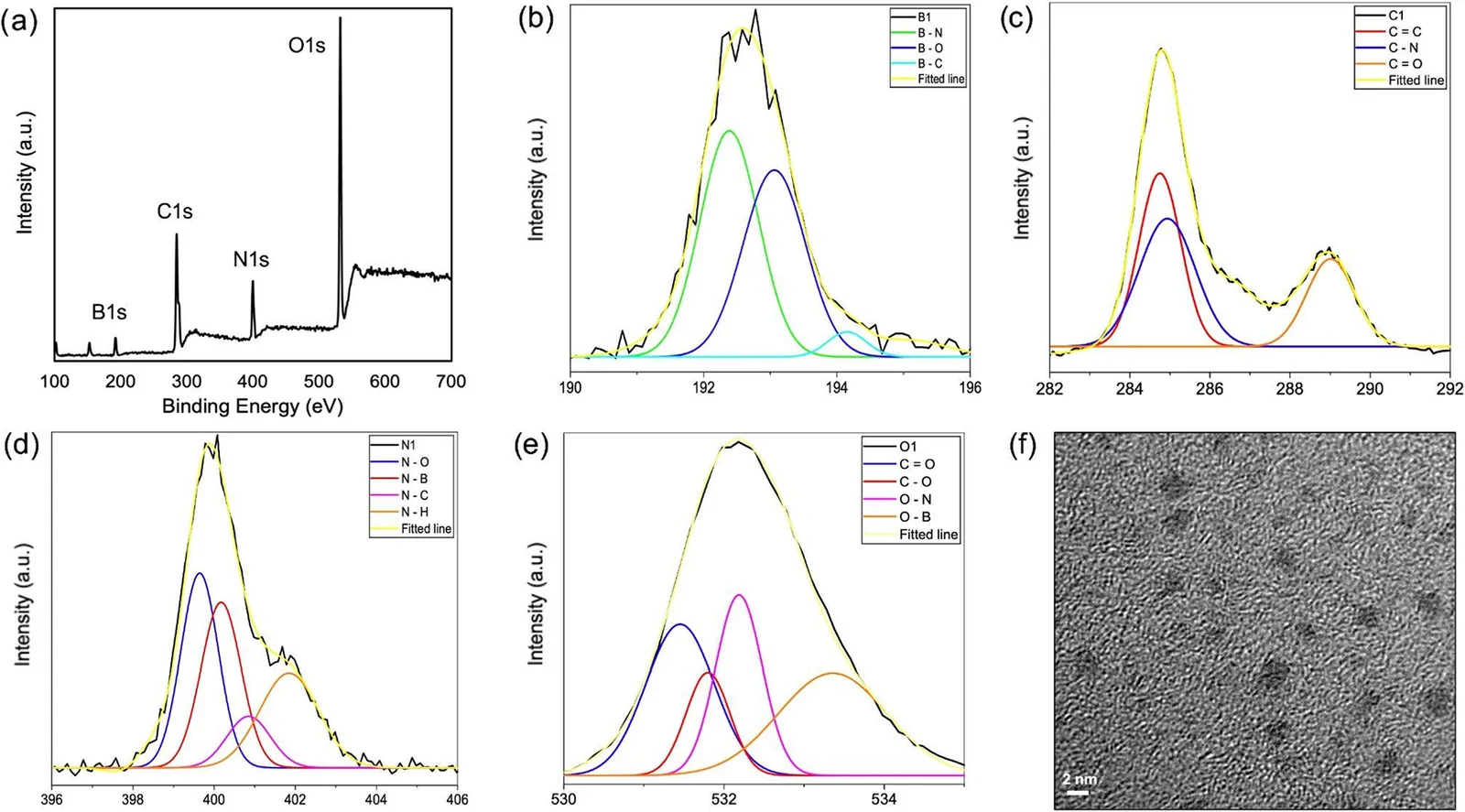
(a) XPS survey of B-CDs, high-resolution XPS survey of (b) B 1s, (c) C 1s, (d) N 1s, (e) O 1s of B-CDs, and (f) TEM image of B-CDs.

The high-resolution (HR) B 1s spectrum exhibited distinct peaks centered at 192.3, 193.0, and 194.5 eV, which were assigned to B–N, B_2_O_3_, and B–C bonding configurations, respectively (Figure 4b), indicating both boron incorporation into the carbon framework and the existence of a boron oxide matrix. The HR C 1s spectrum could be deconvoluted into three main components corresponding to C–C/C=C (285.0 eV), C–N/C=N (285.1 eV), and C=O (289.0 eV) bonds, reflecting the coexistence of graphitic carbon domains and surface oxygen/nitrogen-containing functional groups^32^ (Figure 4c).

Similarly, the HR N 1s spectrum revealed multiple nitrogen-bonding environments, including peaks assigned to N–B (400.0 eV), graphitic N–C (401.2 eV), and N–H (402.0 eV), suggesting successful nitrogen incorporation via urea-derived functionalities (Figure 4d). The O 1s spectrum displayed several characteristic components at approximately 531.0, 532.0, 532.4, and 534.0 eV, corresponding to C=O, O–H/C–O, O=C–O, O–N, and O–B functionalities, respectively (Figure 4e), further confirming the presence of oxygen-rich surface groups and boron–oxygen interactions.^33^

Overall, the XPS results are in good agreement with the FTIR analysis, supporting the formation of B-CDs. The presence of B–C and B–N bonding indicates chemical interactions between boric acid and the carbon precursors during thermal treatment, while the B_2_O_3_-related component suggests the presence of a boron-rich environment surrounding the carbonaceous domains. These findings suggest that the boric acid-derived environment may contribute to both structural confinement and the modification of the surface chemical states of the CDs, which may collectively favor solid-state photoluminescence. These structural features are expected to contribute significantly to the enhanced solid-state fluorescence and stability of the synthesized B-CDs. As shown in Figure 4f, TEM characterization revealed uniformly distributed, quasi-spherical B-CDs with an average particle size of 4.7 ± 1.0 nm. The absence of significant aggregation indicates effective control over nanoparticle formation during synthesis. Additional TEM images and the corresponding particle size distribution are presented in Figure S3.

### 3.1 Stability

The photoluminescence (PL) stability of the synthesized B-CDs was systematically evaluated under moisture, thermal, ambient air, and ultraviolet (UV) irradiation conditions. To investigate moisture resistance, the B-CDs were exposed to an environment with 95% relative humidity for 60 min. The PL measurements showed that more than 95% of the initial emission intensity was preserved after 1 h of exposure to 95% relative humidity, demonstrating strong short-term moisture stability.

The thermal stability of the B-CDs was further examined under different temperatures. The PL intensity showed negligible degradation after short-term heating treatments, and the emission efficiency remained above 95% even at 90 °C. This enhanced thermal robustness is attributed not only to the intrinsic stability of the carbonized structure but also to the rigid boric acid matrix, which effectively suppresses nonradiative relaxation and protects the emissive centers under thermal stress.

Long-term ambient stability is particularly important for practical solid-state fluorescent applications. As shown in Figure 5, the B-CD powders retained more than 95% of their original PL intensity after storage under room-temperature atmospheric conditions for 30 days, indicating good ambient stability. In addition, photostability under continuous UV exposure is critical for forensic fingerprint visualization applications. Therefore, the B-CDs were continuously irradiated with UV light for 60 min, during which no noticeable decrease in PL intensity was observed, corresponding to nearly 100% PL retention. These results demonstrate the remarkable environmental stability of the synthesized B-CDs and highlight their strong potential for practical solid-state and forensic applications.

**Figure 5:**
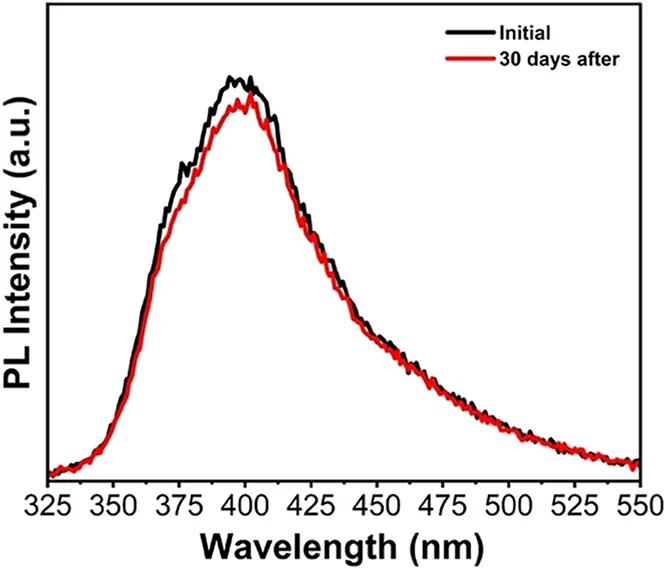
Long-term ambient stability of B-CD powders under room-temperature storage, retaining over 95% of the initial PL intensity after 30 days.

The environmental stability of the synthesized powder B-CDs was evaluated under humid conditions, UV irradiation (365 nm), and elevated temperatures, as shown in Figure 6. Under humid conditions, the PL spectra exhibited negligible changes over 1 h, retaining approximately 98–100% of the initial intensity (Figure 6a), demonstrating strong short-term moisture stability. During prolonged exposure, the B-CDs retained approximately 57% of their initial PL intensity after 5 days under 95% relative humidity, indicating a gradual decrease in fluorescence under long-term humid conditions (Figure S4a). Similarly, under continuous 365 nm UV exposure, the B-CDs maintained nearly unchanged emission profiles and relative PL intensity above 97% after 1 h (Figure 6b), demonstrating strong short-term photostability. After 5 days of continuous UV irradiation, the PL intensity remained at approximately 92% of its initial value, indicating strong long-term photostability (Figure S4b). The thermal stability results revealed that the B-CDs preserved their fluorescence up to 90 °C with minimal intensity loss during short-term exposure (Figure 6c). However, a significant decrease in PL intensity (∼60%) was observed at 120 and 150 °C, likely due to thermally induced changes in surface functional groups and increased nonradiative recombination. Long-term thermal aging at 50 °C retained approximately 48% of the initial PL intensity after 5 days, indicating a pronounced effect of prolonged thermal exposure on fluorescence stability (Figure S4c). Overall, these results reveal a clear distinction between the short-term and long-term stability of the B-CDs. While strong stability was observed during short-term humidity, UV, and thermal exposure, prolonged humidity and thermal aging resulted in greater PL intensity losses, whereas the B-CDs maintained comparatively strong long-term photostability under continuous UV irradiation. The corresponding variations in PL peak intensity during the humidity, UV exposure, and temperature stability tests are provided in the Supporting Information (Figure S5a–c).

**Figure 6:**
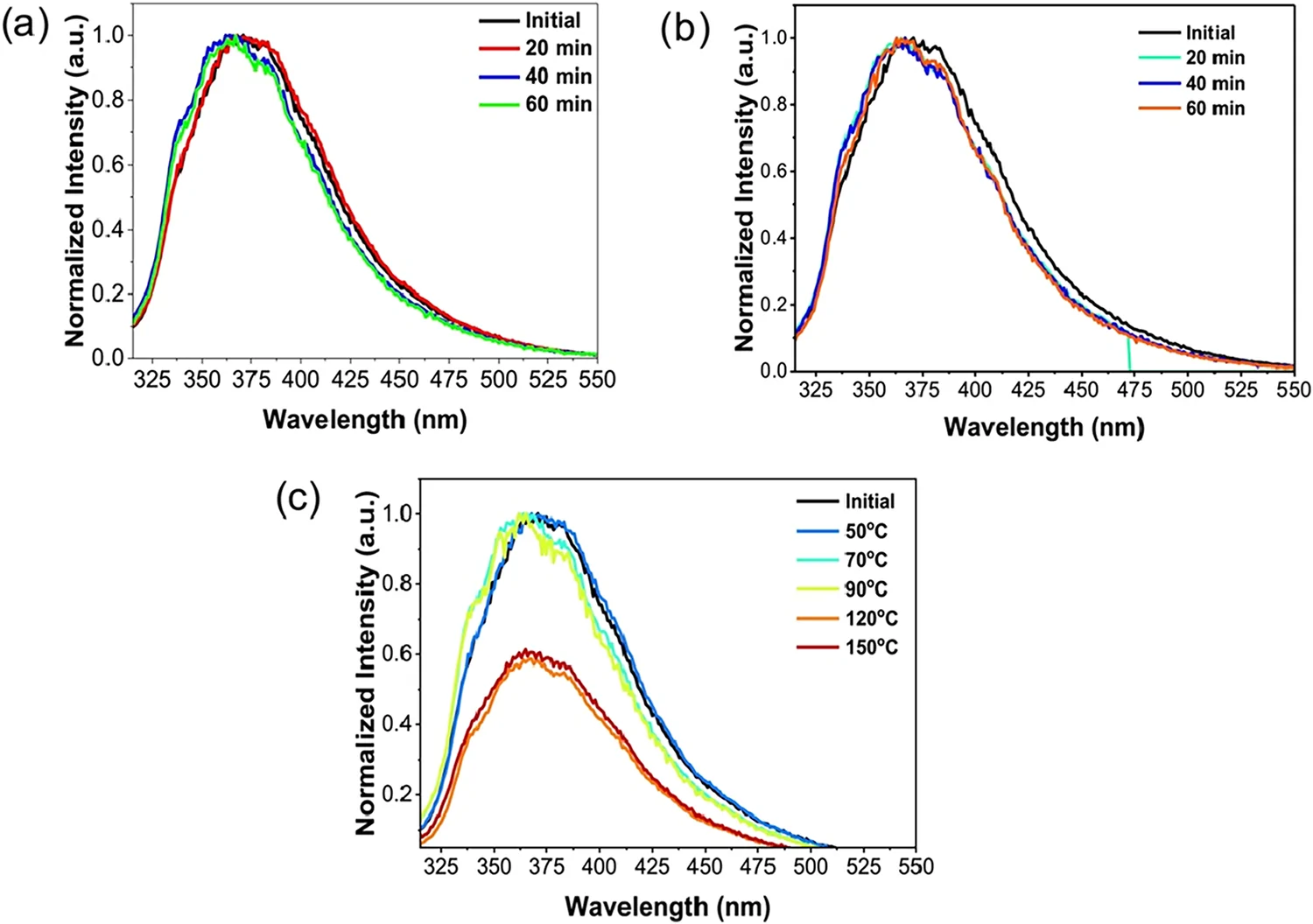
(a) Stability of the normalized photoluminescence (PL) intensity under different relative humidity conditions, (b) stability of the normalized PL intensity over time under 365 nm UV irradiation, and (c) PL intensity of powdered B-CD pellets measured at different temperatures.

### 3.2 Latent Fingerprint Visualization and Practical Applicability

To evaluate the practical applicability of the synthesized B-CDs in forensic investigations, their performance in LFP visualization was systematically examined, leveraging their optical properties and favorable surface chemistry. In this study, glass (lamella) was selected as the primary substrate, and blue–green, fluorescent B-CD powders were applied to the surface. Fingerprints were obtained from volunteer donors following the protocol described in the Section 2. As illustrated in Figure 8, the developed fingerprint on the glass surface exhibited clearly distinguishable ridge patterns and characteristic fingerprint features. Level I features, including overall ridge flow patterns and characteristic structures such as whorls (1), deltas, and lakes (2), were distinctly observed. More importantly, Level II minutiae, which are crucial for individual identification, such as ridge endings (4) and bifurcations (5), were well-resolved.^34^ In addition, enlarged fluorescence images revealed selected Level III features, particularly sweat pores, along the fingerprint ridges, demonstrating the capability of the B-CD powder to visualize fine fingerprint details.^35^ The effective visualization of fingerprint ridge features may be facilitated by the presence of abundant surface functional groups (−COOH, −NH_2_, and −OH) on the CDs, which can promote interactions with organic and inorganic constituents of fingerprint residues, such as lipids, amino acids, and salts, through hydrogen bonding and electrostatic interactions.^36^ Furthermore, the CD-based method exhibited clear ridge-to-background differentiation in the fluorescence images, as supported by quantitative R/B intensity analysis.^37^ The performance characteristics of the proposed B-CDs were compared with previously reported carbon-dot-based fingerprint visualization systems in terms of synthesis conditions, quantum yield, detectable fingerprint features, and stability, as summarized in Table 1. However, direct comparison of quantum yield values across studies should be interpreted with caution, as the reported values may have been obtained under different measurement conditions. To assess durability, the developed samples were stored at room temperature for 30 days under ambient conditions. The results indicated that both fluorescence intensity and structural integrity of the fingerprint features were well preserved over time, confirming the long-term stability and practical potential of CDs for forensic applications.

**Table 1:** Comparison of the Present B-CD System with Previously Reported Carbon-Dot-Based Materials for Latent Fingerprint Visualization^a^

ref	Material	Production Method	PLQY (%)	PLQY Excitation Wavelength (nm)	Levels	Stability
Dong et al.^35^	R-CDs-Starch	Hydrothermal (180 °C 4 h)	21	550	Level III	Not reported
Niu et al.^17^	CDs	Open Reactor (200 °C 3 h)	18.2	540	Not reported	Photostability (60 min) and Air stability (30 days)
Ding et al.^37^	Fe_3_O_4_@CDs	Hydrothermal (200 °C 2 h)	45.04	370	Level III	Not reported
Lv et al.^21^	EDF_CDs_Starch	Alkaline Condensation (5 days)	Not reported	365, 390, 415, 450	Level III	Not reported
Li et al.^38^	FNCDs-Starch	Microwave (650w 5 min)	26.7	407	Level III	Not reported
You et al.^39^	CD@mSiO_2_	Hydrothermal (120 °C 4 h)	41.77	360	Level III	Not reported
Lotey et al.^40^	N-CD	Hydrothermal (200 °C 4 h)	19.73	420	Level III	Air stability (days)
Lighvan et al.^41^	N–Fe_CD-Starch	Microwave (900w 4 min)	11.3	450	Not reported	Not reported
Jabeen et al.^32^	Y-CDs	Microwave (700w 3 min)	22.5	520	Level III	Not reported
Cui et al.^42^	CDs	Hydrothermal (200 °C 4 h)	3.24	265	Level III	Air stability (6 months) and Photostability (10 days)
This Work	B-CDs	Solvent-free thermal synthesis (160 °C, 1 h, N_2_)	77.5	370	Level II and selected Level III	Air stability (30 days), Photostability (5 days), Thermal stability (5 days) and Humidity stability (5 days)

a All PLQY values listed in the table correspond to solid-state measurements. The excitation wavelength used for PLQY measurement is provided where available in the original reports. “Not reported” indicates that the corresponding information was not clearly specified in the original study. PLQY values obtained under different excitation wavelengths and experimental conditions should be compared with caution.

Finally, to evaluate the practical applicability of the B-CDs for LFP visualization, the developed powder was tested on six representative everyday surfaces: banknote, aluminum foil, glass, plastic, porcelain, and metal. Representative fingerprints developed on banknote, aluminum foil, and glass are presented in Figure 7 under daylight and UV (365 nm) illumination. To further demonstrate the applicability of the B-CD powder to different substrate types, additional fingerprint images obtained on plastic, porcelain, and metal surfaces are provided in Figure S6. Clear fluorescent ridge patterns were observed on all tested substrates under UV illumination, demonstrating the applicability of the B-CD powder across surfaces with different physical and chemical characteristics.

**Figure 7:**
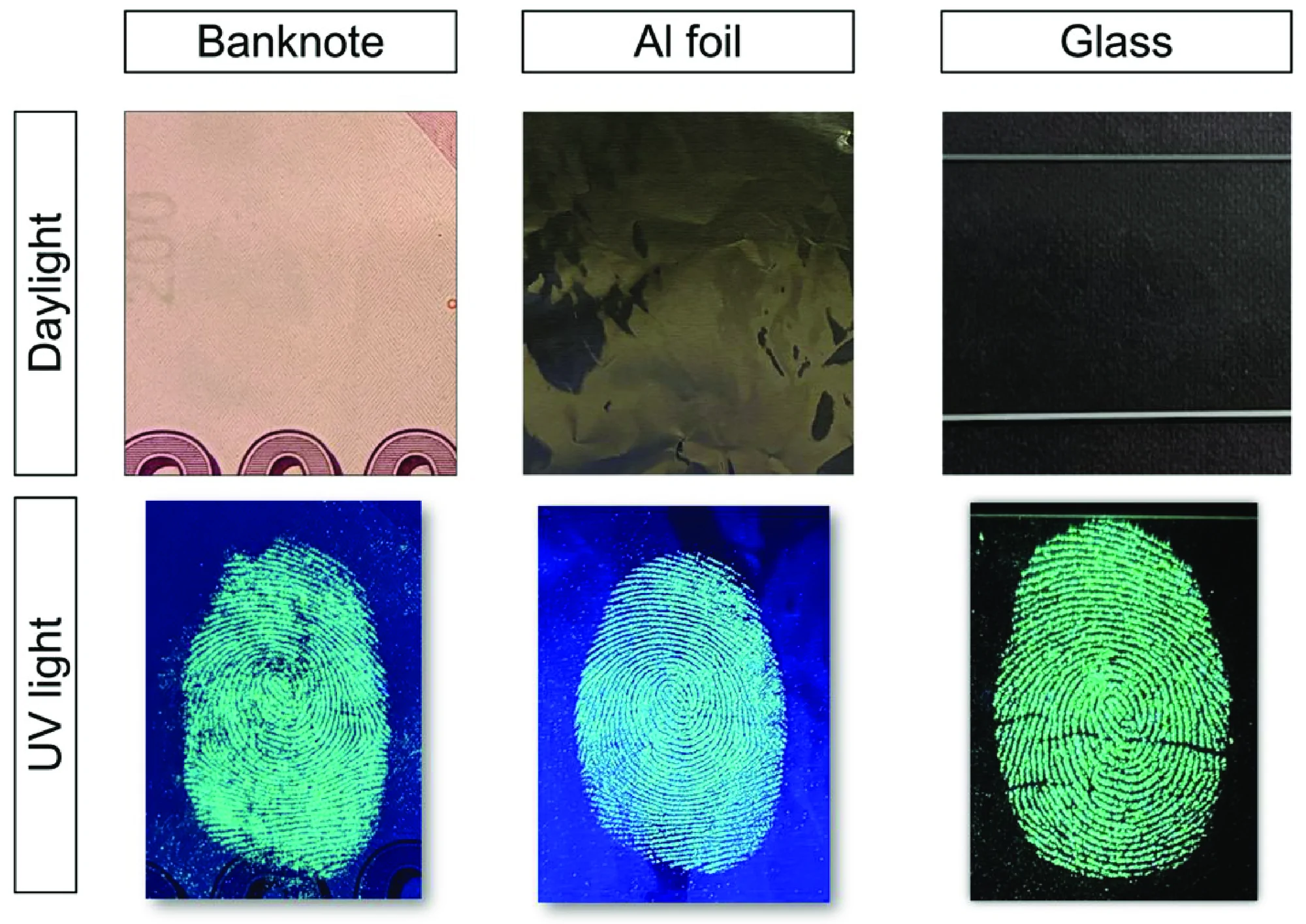
Representative latent fingerprints developed with B-CD powder on banknote, aluminum foil, and glass under daylight and UV (365 nm) illumination.

Detailed analysis of the fingerprints revealed that Level II features, including ridge endings and bifurcations, were clearly distinguishable across the tested substrate types. As illustrated in Figure 8, additional fingerprint characteristics, including cores, bridges, and crossover structures, could also be identified. Enlarged fluorescence images further revealed selected Level III features, particularly sweat pores along the fingerprint ridges. Overall, these results demonstrate the capability of the fluorescent B-CD powder to visualize characteristic ridge minutiae and fine fingerprint details.

**Figure 8:**
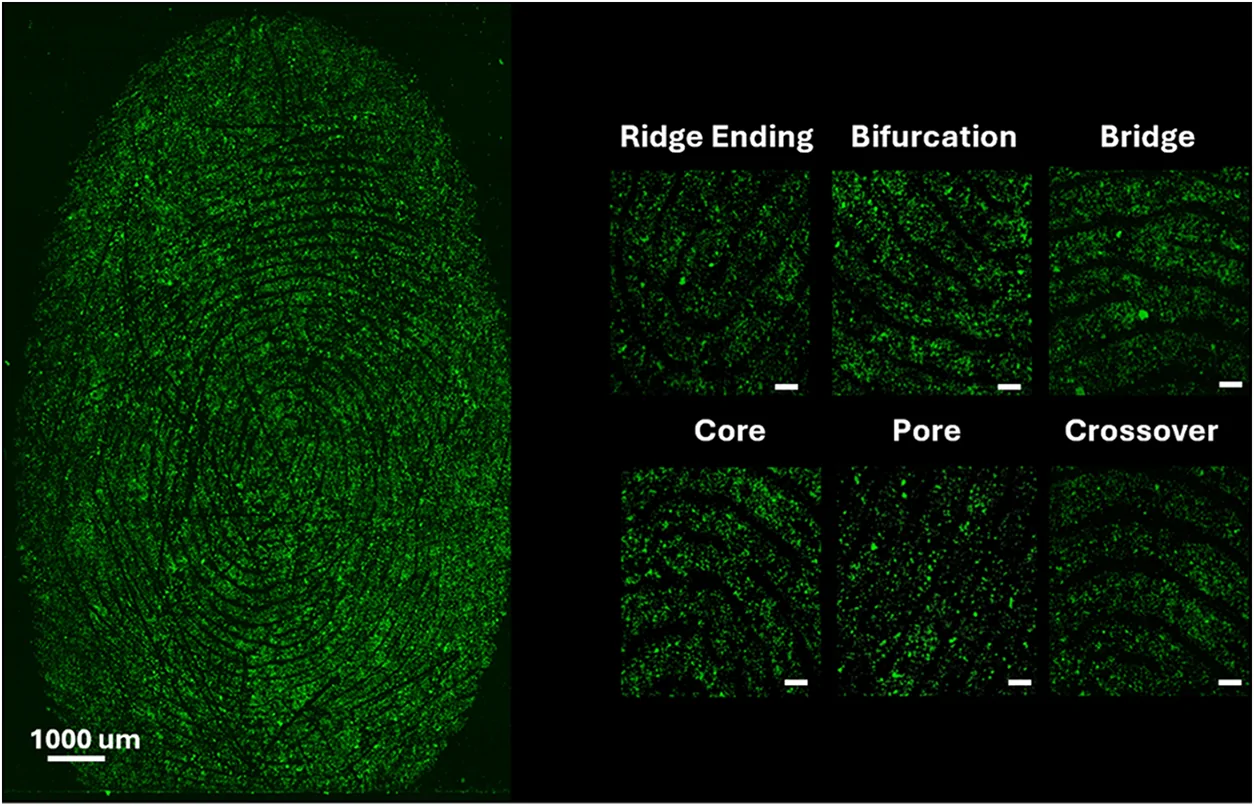
Representative fluorescence image of a latent fingerprint developed using B-CD powder and enlarged views showing characteristic fingerprint features, including ridge ending, bifurcation, bridge, core, crossover, and selected Level III features such as sweat pores. Scale bars: 1000 μm in the full fingerprint image and 200 μm in the enlarged images.

To quantitatively evaluate the ridge-to-background contrast, image-based intensity analysis was performed using five independent ROIs from representative fingerprint images obtained on glass, banknote, and aluminum foil surfaces. The mean R/B ratios were 3.77 ± 0.99 for glass, 3.36 ± 0.94 for banknote, and 4.86 ± 4.09 for aluminum foil (*n* = 5). In all three cases, the ridge intensity exceeded the corresponding local background intensity, quantitatively supporting the clear ridge-to-background differentiation observed in the fluorescence images. The representative ROIs used for the analysis are provided in Figure S7.

## 4 Conclusion

In this study, highly luminescent B-CDs were successfully synthesized via a simple, rapid, and solvent-free thermal method at 160 °C for 1 h under a nitrogen atmosphere. Unlike conventional carbon dot synthesis approaches that often require solvents, purification, or post-treatment procedures, the developed strategy enabled the direct production of strongly emissive solid-state B-CD powders. The synthesized B-CDs exhibited strong solid-state fluorescence with a high PLQY of 77.5% in powder form. Structural and spectroscopic analyses indicated chemical interactions between boric acid and the carbon precursors and supported the contribution of boric-acid-induced structural and surface-state modifications to the enhanced solid-state emission. The enhanced photoluminescence is therefore proposed to arise from the combined effects of structural confinement and surface-state modification rather than from physical confinement alone. Consistent with this interpretation, TRPL analysis revealed an increase in the amplitude-weighted average PL lifetime from 0.1749 ns for pristine CDs to 2.763 ns for B-CDs, indicating altered excited-state relaxation dynamics in the boric-acid-assisted system.

The B-CDs exhibited strong short-term stability, retaining more than 95% of their initial PL intensity after 60 min exposure to 95% relative humidity and during short-term thermal treatment up to 90 °C. Long-term measurements revealed condition-dependent stability, with approximately 57% PL retention after 5 days at 95% relative humidity and approximately 48% after 5 days of thermal aging at 50 °C, while approximately 92% of the initial PL intensity was retained after 5 days of continuous UV irradiation. The prepared B-CDs were successfully applied as fluorescent powders for latent fingerprint visualization on various substrates, enabling clear visualization of ridge patterns and characteristic Level II minutiae, including ridge endings and bifurcations. Enlarged fluorescence images additionally revealed fine structural characteristics along selected fingerprint ridges, although these observations were interpreted cautiously rather than as definitive evidence of all Level III features. Overall, these findings demonstrate that boric acid-assisted solvent-free synthesis provides a promising strategy for developing highly emissive solid-state carbon dots and highlight their potential for fluorescence-based latent fingerprint visualization.

## Supplementary Material



## Notes

Photographs of pristine carbon dots synthesized without boric acid under daylight and UV illumination; comparative TRPL decay curves, fitting parameters, and PL emission spectra of pristine CDs and B-CDs; supplementary TEM images and particle size distribution of B-CDs; short- and long-term photoluminescence stability measurements under humidity, UV irradiation, and thermal conditions; additional latent fingerprint images on representative substrates; and quantitative ridge-to-background fluorescence intensity analysis (PDF)
