# Supplementary material for: Solvent-Free Synthesis of Highly Luminescent Solid-State Boric Acid-Assisted Carbon Dots for Stable and High-Contrast Latent Fingerprint Visualization
Source: ACS Omega. 2026 Oct 20;11(43):67422–32. doi: 10.1021/acsomega.6c08569 (PMC13737954; doi:10.1021/acsomega.6c08569)
Supplement: Supplementary file 1 [file ao6c08569_si_001.pdf]

Supporting Information

Solvent-Free Synthesis of Highly Luminescent Solid-State Boric Acid-Assisted Carbon Dots for Stable and High-Contrast Latent Fingerprint Visualization

Sema Karabel Ocal[1,2], Kevser Sahin Tiras[3,*], Evren Mutlugun[1,2,4*]

[1] Nanome Nanotechnology Co., Kayseri 38140, Türkiye
[2] Department of Electrical-Electronics Engineering, Abdullah Gul University, Kayseri 38080, Türkiye
[3] Department of Physics, Erciyes University, Kayseri 38030, Türkiye
[4] UNAM—Institute of Materials Science and Nanotechnology Bilkent University, Bilkent, Ankara, Türkiye

∗ Authors to whom any correspondence should be addressed.
E-mail: kevsersahintiras@erciyes.edu.tr and evren.mutlugun@agu.edu.tr

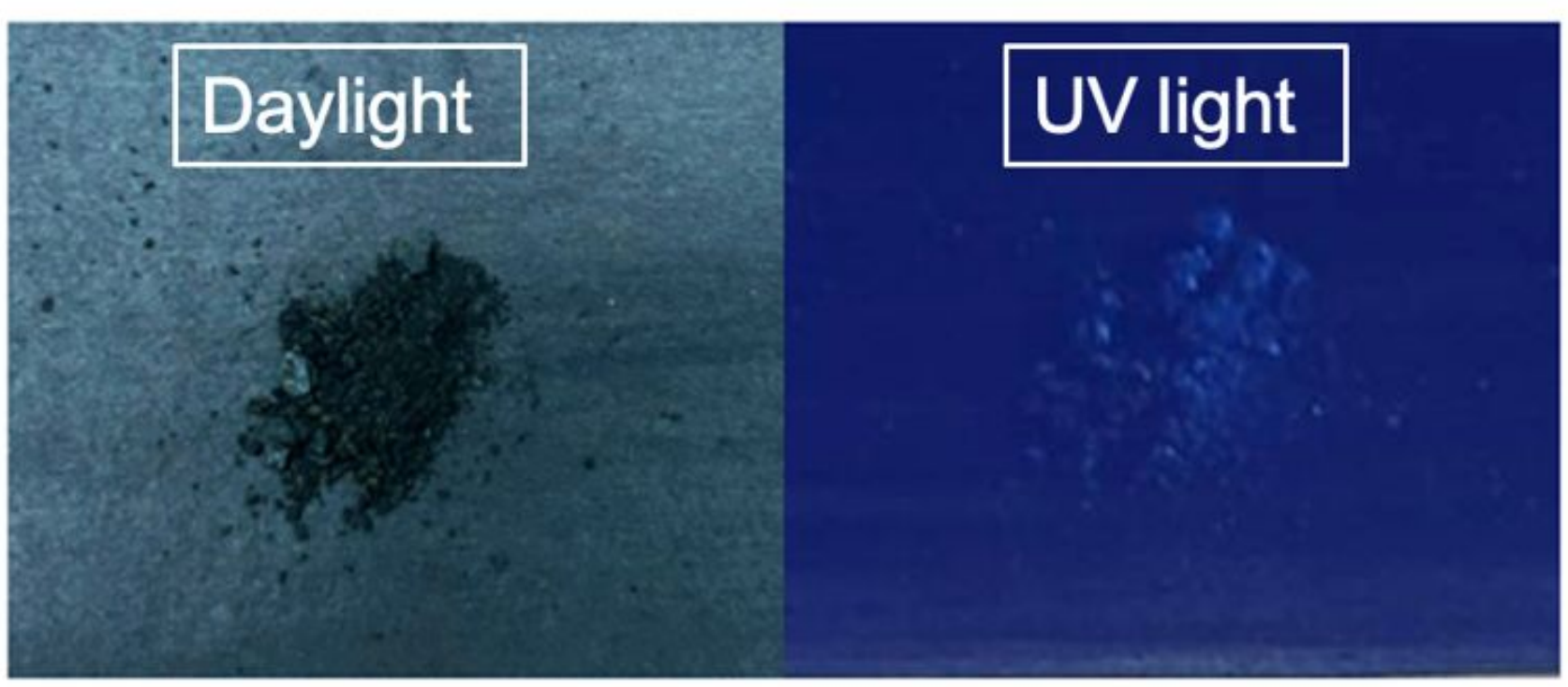

Figure S1. Photographs of pristine carbon dot powder synthesized without boric acid under daylight and UV illumination, showing negligible visible solid-state fluorescence under UV excitation.

Table S1. Fitted TRPL decay parameters and amplitude-weighted average lifetimes of pristine CDs and B-CDs measured under 340 nm excitation.

	**CD**	**B-CD**
$\mathbf{A_1}$	8567± 883	1668.7 ± 43.5
$\boldsymbol{\tau_1}$ **(ns)**	0.1193± 0.0124	11.595 ± 0.211
$\mathbf{A_2}$	824 ± 142	2235 ± 145
$\boldsymbol{\tau_2}$ **(ns)**	0.7523 ± 0.0886	2.917 ± 0.192
$\mathbf{A_3}$	-	6172 ± 670
$\boldsymbol{\tau_3}$ **(ns)**	-	0.2898 ± 0.0388
$\boldsymbol{\tau_{average}}$ **(ns)** **(amplitude weighted)**	**0.1749**	**2.763**

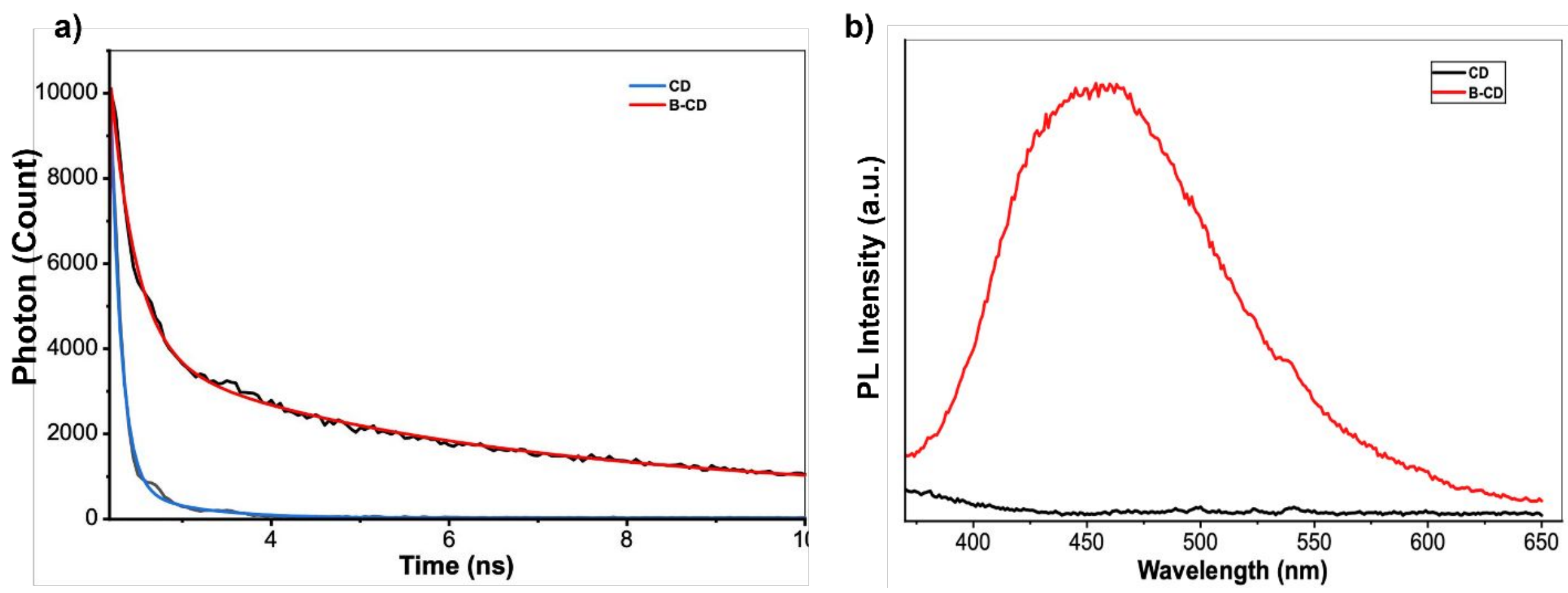

Figure S2. Comparison of the photophysical properties of pristine CDs and B-CDs: **(a)** TRPL decay curves measured under 340 nm excitation and **(b)** PL emission spectra of pristine CDs and B-CDs.

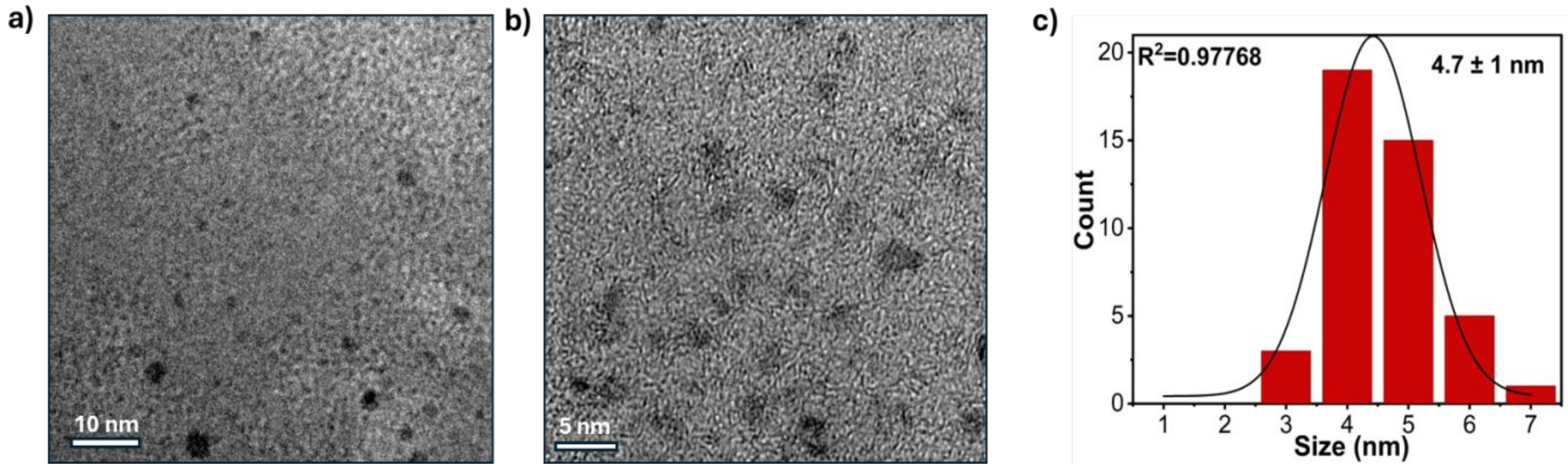

Figure S3. (a,b) Representative TEM images of B-CDs dispersed in deionized water and (c) corresponding particle-size distribution histogram.

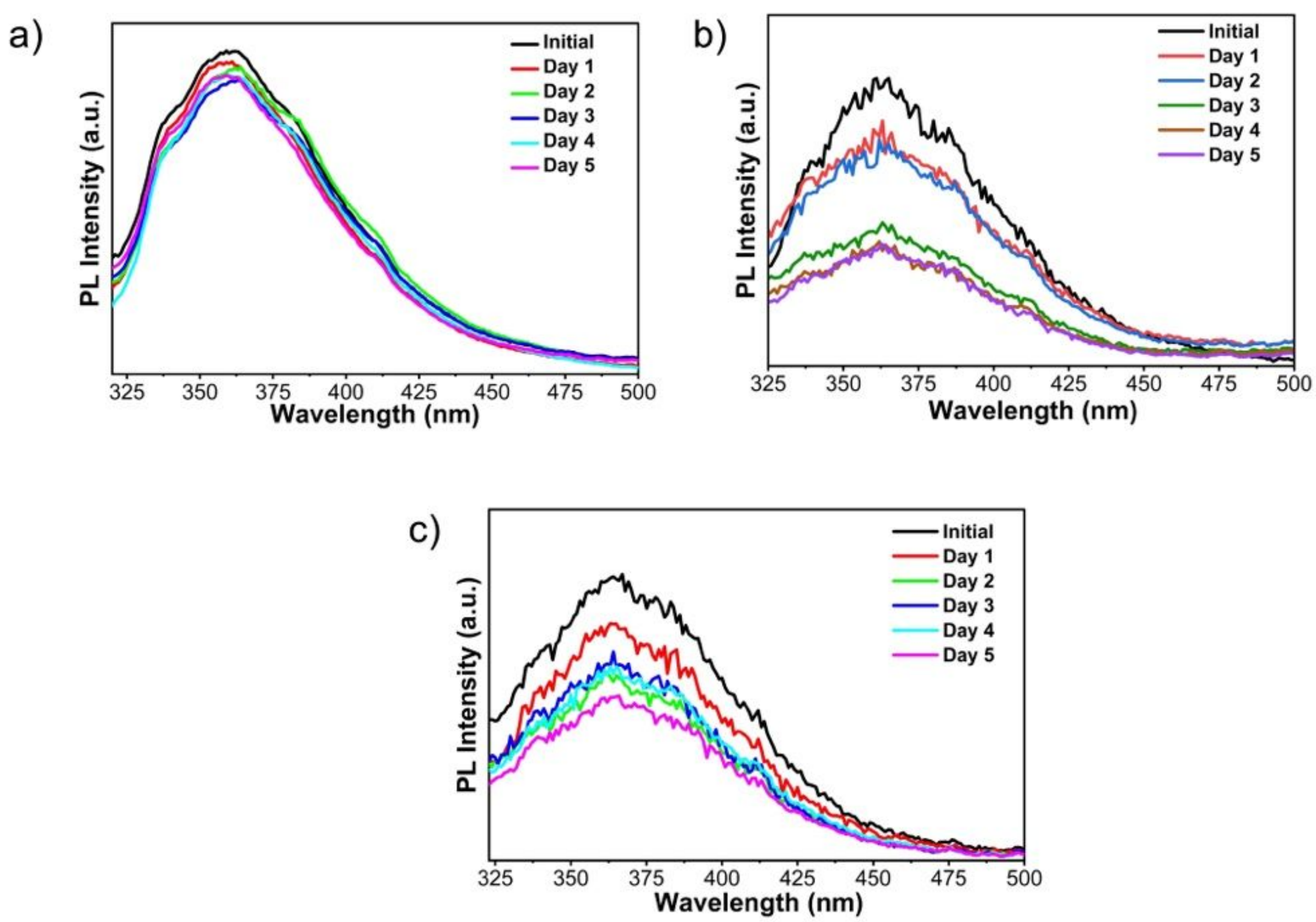

Figure S4. Relative photoluminescence (PL) intensity retention of the synthesized B-CDs during long-term stability tests under (a) 95% relative humidity, (b) continuous 365 nm UV irradiation, and (c) thermal aging at 50 °C over a period of 5 days.

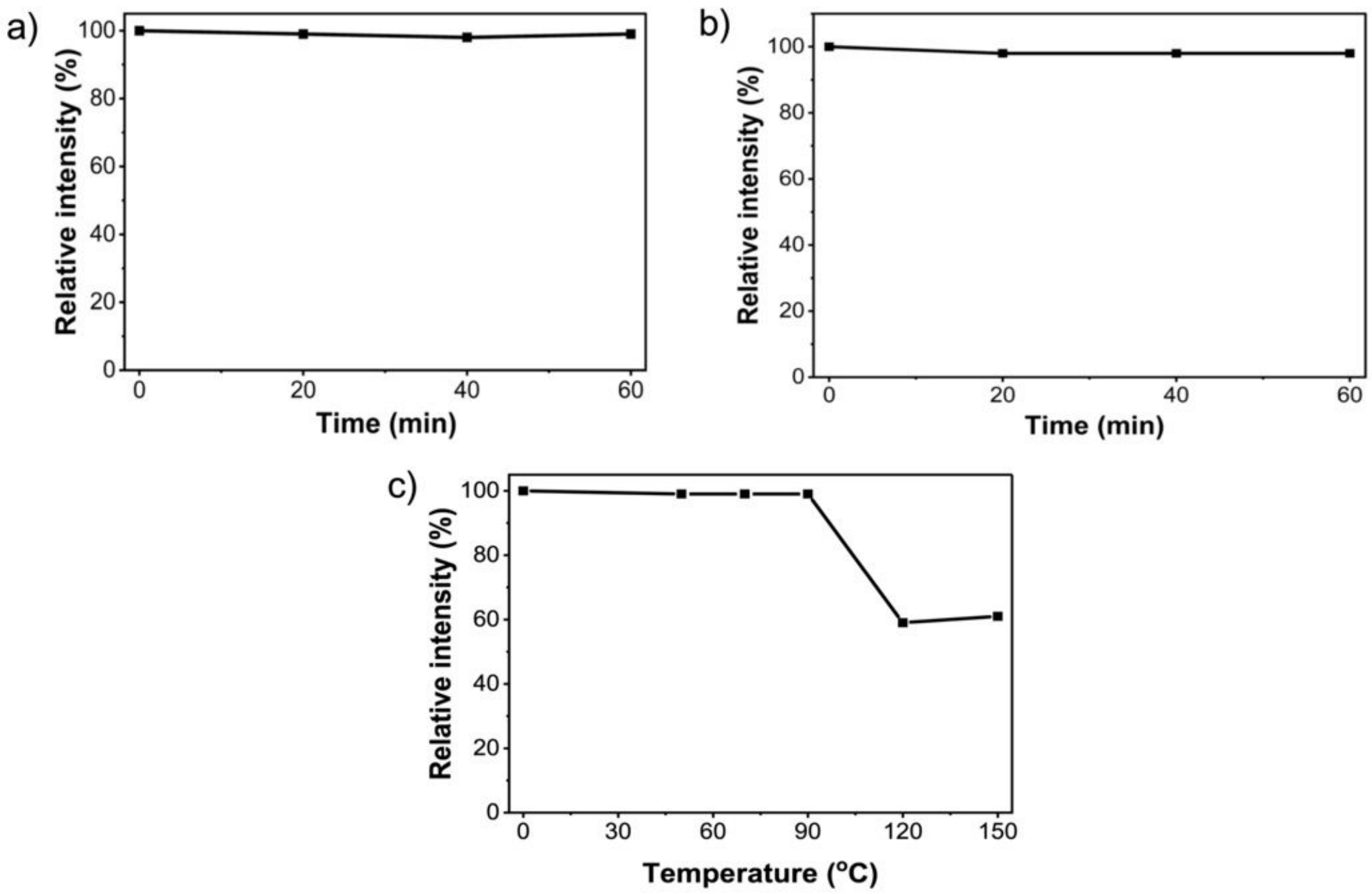

Figure S5. PL peak intensity during the humidity (a), UV exposure (b), and temperature (c) stability tests.

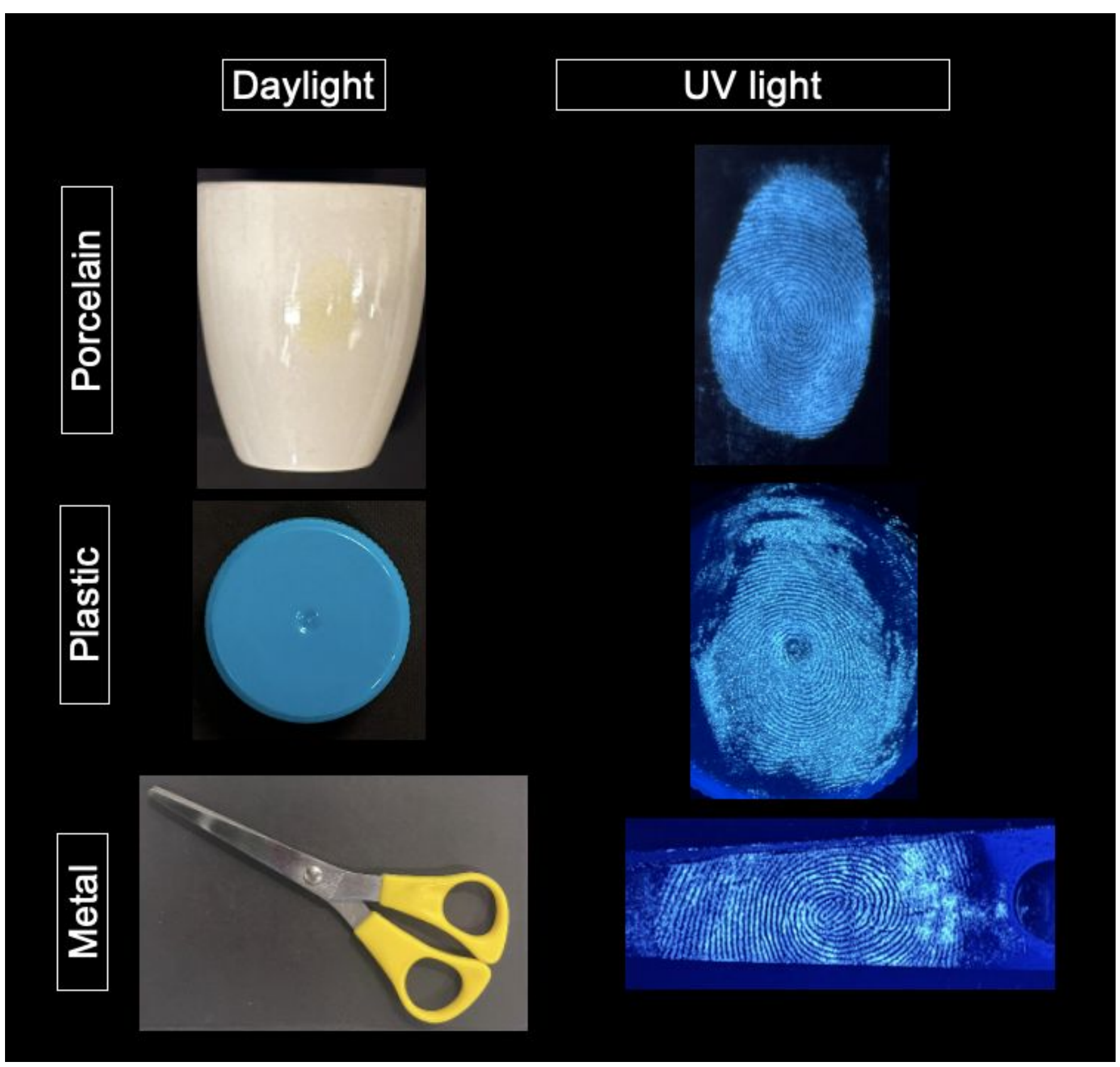

Figure S6. Representative photographs of latent fingerprints developed with B-CD powder on porcelain, plastic, and metal surfaces under daylight and UV (365 nm) illumination. The fluorescence images clearly visualize fingerprint ridge patterns on substrates with different surface characteristics.

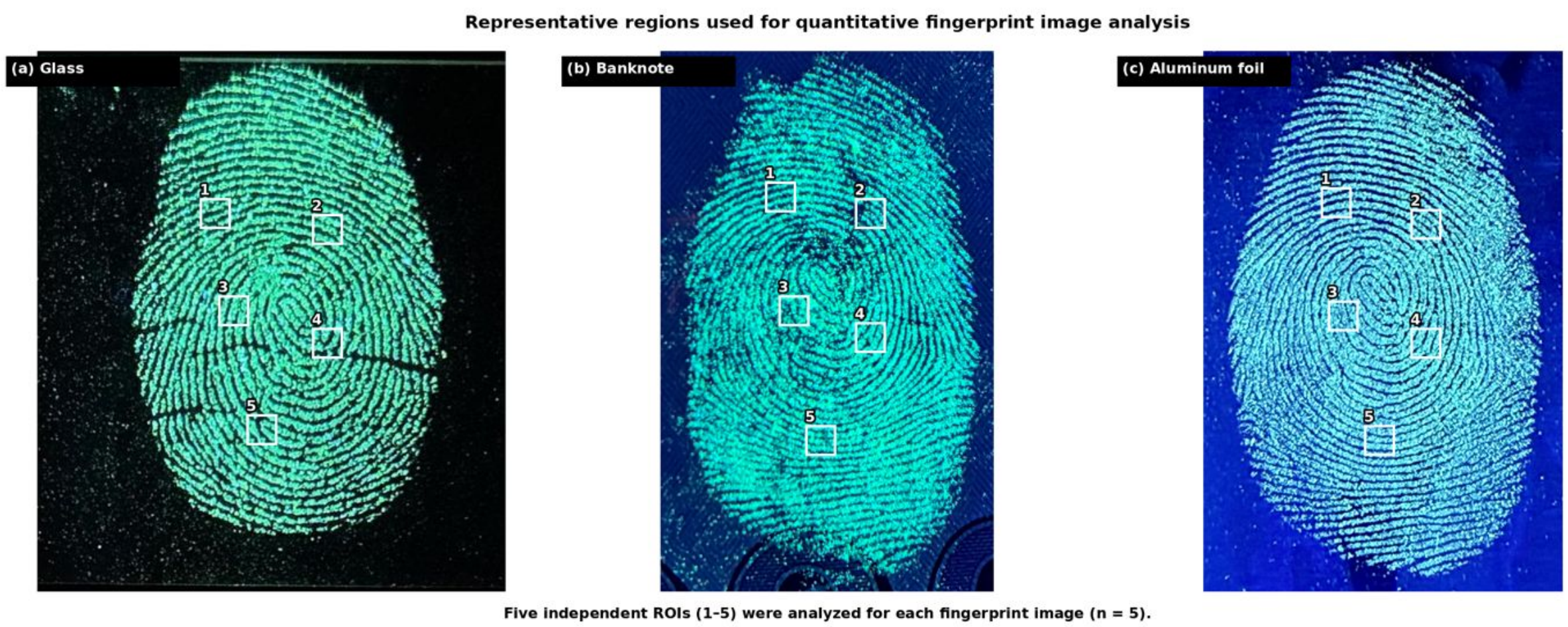

Figure S7. Representative regions of interest (ROIs) used for quantitative ridge-to-background fluorescence intensity analysis of latent fingerprints developed on (a) glass, (b) banknote, and (c) aluminum foil surfaces. Five independent ROIs were evaluated for each image (n = 5).
